# 三聚氰胺功能化多孔有机聚合物的合成及其对甲基橙的吸附性能

**DOI:** 10.3724/SP.J.1123.2021.06016

**Published:** 2021-09-08

**Authors:** Chong ZHANG, Yun GUO, Zifang PENG, Wenfen ZHANG, Shusheng ZHANG

**Affiliations:** 1.郑州大学化学学院, 河南 郑州 450001; 1. College of Chemistry, Zhengzhou University, Zhengzhou 450001, China; 2.郑州大学现代分析与基因测序中心, 河南 郑州 450001; 2. Center of Advanced Analysis and Gene Sequencing, Zhengzhou University, Zhengzhou 450001, China

**Keywords:** 多孔有机聚合物, 席夫碱反应, 吸附, 三聚氰胺, 甲基橙, porous organic polymers, Schiff-base reaction, adsorption, melamine, methyl orange (MO)

## Abstract

首先以对三联苯(TP)和对苯二甲酰氯(TC)为单体合成了酮基聚合物前体(TP-TC),而后基于席夫碱反应将三聚氰胺(MA)与之交联得到胺功能化的多孔有机聚合物TP-TC-MA。通过扫描电镜(SEM)、透射电镜(TEM)、傅里叶变换红外光谱(FT-IR)、X射线衍射(XRD)、氮气吸附-脱附(BET)以及零电荷点(pH_pzc_)的测定等手段对合成的多孔有机聚合物进行表征。以染料废水中典型的阴离子染料甲基橙为研究对象,研究了TP-TC-MA对其吸附行为,并探讨了吸附机制。利用紫外-可见分光光度计(UV-Vis)得出目标污染物的紫外吸收光谱标准曲线,标准曲线拟合相关系数(*R*^2^)为0.999。结果表明,TP-TC-MA由于具有高比表面积(708.5 m^2^/g)和总孔体积(0.556 cm^3^/g)以及丰富的含氮基团,对阴离子染料甲基橙表现出优异的吸附性能。TP-TC-MA对水中甲基橙的吸附动力学符合准二级动力学方程,吸附平衡数据可以采用Langmuir等温吸附模型描述。经由Langmuir等温吸附模型计算得到的理论最大吸附容量为156.3 mg/g。选择性实验结果表明,TP-TC-MA对阴离子染料甲基橙具有更强的吸附作用,吸附作用机理可归结为静电相互作用、氢键和*π-π*相互作用等。在重复使用5次之后,TP-TC-MA对甲基橙仍保持90%以上的去除率,表明材料具有良好的稳定性和重复利用性,在染料废水处理中具有很好的应用前景。

## Abstract (en)

In this work, a polymer precursor was first synthesized using *p*-terphenyl (TP) and terephthaloyl chloride (TC) as monomers. Then, cross-linking was realized by means of a Schiff base reaction with melamine (MA) as a modifier to obtain an amine-functionalized porous organic polymer TP-TC-MA. The synthesized polymers were characterized by scanning electron microscopy (SEM), transmission electron microscopy (TEM), Fourier transform infrared (FT-IR) spectroscopy, X-ray diffraction (XRD), and point of zero charge (pH_pzc_) measurements, as well as on the basis of nitrogen adsorption-desorption isotherms. Adsorption experiments were carried out to evaluate the adsorption properties of TP-TC-MA for methyl orange (MO), a typical anionic azo dye that has widespread industrial application. The amount of MO adsorbed on TP-TC-MA was evaluated by ultraviolet-visible (UV-Vis) spectroscopy at a wavelength of 463 nm. Microscopic analysis revealed that the as-synthesized polymer had an aggregated particle-shaped structure. XRD spectra confirmed that TP-TC-MA was an amorphous polymer, consistent with the results of high-resolution TEM experiments. The Brunauer-Emmett-Teller (BET) specific surface area and total pore volume of TP-TC-MA were determined as 708.5 m ^2^/g and 0.556 cm^3^/g, respectively. The measured pH_pzc_ of TP-TC-MA was 4.0, probably because of the abundant nitrogen-containing groups provided by MA. The factors affecting adsorption, such as pH, adsorbent dosage, contact time, initial pollutant concentration, and ionic strength, were investigated. Because of the protonation of the N-atom in TP-TC-MA, the pH had a strong impact on the adsorption of MO. The removal efficiency could be maximized at the optimized pH of 3.0. The adsorption equilibrium isotherm, measured at 25 ℃ and a concentration of 50-500 mg/L, showed that the MO adsorption over TP-TC-MA followed the Langmuir isotherm, with a maximum adsorption capacity of 156.3 mg/g. The modeling of the experimental adsorption data was consistent with the pseudo-second-order kinetic model, which indicated fast adsorption and chemisorption as the dominant mechanism. With increasing ionic strength, the adsorption of MO slightly decreased, suggesting a partial antagonistic ion effect. Results of the selectivity study revealed that TP-TC-MA was more selective toward MO than methylene blue (MB), which indicated that electrostatic interactions played a significant role during the adsorption progress. Five adsorption-desorption cycles showed that TP-TC-MA could be regenerated without significant deterioration of its adsorption efficiency, indicating that it has good stability and reusability. The observed adsorption performance indicated that this MA-modified porous organic polymer offers prospects for further research and application in the treatment of dye-containing wastewaters.

随着印刷和着色工业化过程的不断发展,染料废水带来的环境污染问题日益突出。在染色过程中,尽管可以通过调节电解质来促进染料的吸附着色,但15%~20%的染料仍会由于没有完全吸附在着色品上而随废水排放到环境中,对环境造成严重的危害^[1,2,3]^。染料废水的不当排放不仅会降低水的质量,还会通过氧化、羟基化或其他化学反应而产生有毒化合物^[4]^。偶氮染料是使用最为广泛的一类合成染料,在纺织工业中60%~70%的染料为偶氮染料,包括苏丹红、酸性红、甲基橙、亚甲基蓝等^[5,6]^。这些偶氮染料通常具有稳定的芳香结构,难以通过光降解、氧化和生物降解的途径消除,并且对生物体有致癌和致突变作用。因其导致的环境水体污染将对生态环境产生严重危害^[7,8]^。因此,如何从废水中去除这些染料是亟待解决的环境问题之一。目前,已经有多种方法用以处理染料废水,包括光催化降解、膜过滤、高级氧化法、生物降解法等^[9,10,11,12]^。然而这些方法往往成本高昂,且会造成二次污染。吸附法由于设备简单、能耗低等特点,因此被认为是从废水中去除染料的最有效方法之一^[13]^。

多孔有机聚合物是由有机结构单元设计合成的具有超高交联性的聚合物,包括固有微孔性聚合物(PIM)、多孔有机骨架(POF)、共轭微孔聚合物(CMP)和多孔芳香骨架(PAF)等^[14,15]^。多孔有机聚合物作为一类新兴的高度无定形多孔材料,由于具有较高的表面积、可调的孔径尺寸以及可被后修饰功能化等诸多优点,已经在气体分离与储存^[16]^、色谱分离^[17,18]^、催化^[19]^、污染物去除^[20,21]^、能源^[22]^、化学传感^[23]^等方面被广泛应用。近年来,多孔有机聚合物材料也被广泛用于水中染料分子的吸附去除^[24,25]^。基于聚合物中某些官能团与目标污染物的特定相互作用,功能化后的多孔有机聚合物还显示出对水中染料分子的高效选择吸附^[26,27,28]^。例如,Chen等^[29]^设计合成了一种新型阳离子多孔有机聚合物C-NSA_Naph_HCP@Br,该材料对水中阴离子染料和$Cr_{2}O_{7}^{2-}$的吸附表现出快速、高效、优异的选择性以及良好的可重复利用性。三聚氰胺(MA)具有丰富的氮原子,将其作为后修饰剂引入聚合物可以提高材料含氮量以及亲水性^[30]^。同时,氨基的存在可以显著增强主体分子对阴离子染料的静电吸附作用,进而大大提升吸附剂的吸附性能。

本文以对三联苯(TP)和对苯二甲酰氯(TC)为反应单体合成了酮基聚合物前体TP-TC,而后以三聚氰胺为刚性交联剂基于席夫碱反应成功制备了胺功能化的多孔有机聚合物材料TP-TC-MA,并使用扫描电镜、透射电镜、傅里叶变换红外光谱、氮气吸附-脱附等手段对其进行了表征。以典型的阴离子偶氮染料甲基橙(MO)为目标物考察了材料的吸附性能,并探究了甲基橙溶液初始pH值、材料用量、吸附时间、初始浓度等因素对去除率(*R*)的影响。采用动力学模型和等温线模型对吸附数据进行拟合,并对吸附机理进行了研究。

## 1 实验部分

### 1.1 仪器、试剂与材料

场发射扫描电子显微镜(Auriga, ZEISS,德国);透射电子显微镜(Talos F200S, FEI,美国);红外光谱分析仪(FT-IR Frontier, Perkin-Elmer Inc.,荷兰);氮气吸附-脱附仪(ASAP2420-4MP, Micromeritics,美国);真空干燥箱(DZF-6050,上海一恒科学仪器有限公司);紫外-可见分光光度计(TU-1901,北京普析通用仪器有限公司);超声波清洗仪(KSK 2210LHC,上海科导超声仪器有限公司); pH计(FE20,梅特勒-托力多仪器有限公司,瑞士);纯水机(Milli-Q Integral,Millipore,美国)。

对三联苯、对苯二甲酰氯、三聚氰胺和无水氯化铝购自上海麦克林生化科技有限公司。二氯甲烷、盐酸(纯度36.5%,优级纯)、浓硝酸(纯度63%,优级纯)、氢氧化钠、甲基橙、亚甲基蓝(MB)和无水乙醇、甲醇、二甲基亚砜购自天津科密欧化学试剂公司。除非特殊说明,本工作所用试剂均为分析纯。

### 1.2 三聚氰胺功能化多孔有机聚合物的合成

根据文献^[31]^报道的方法合成三聚氰胺改性的多孔有机聚合物TP-TC-MA。将1.015 g TC(5 mmol)和1.151 g TP(5 mmol)溶解在盛有100 mL二氯甲烷的三口烧瓶中。将溶液保持在20 ℃,并缓慢加入无水AlCl_3_催化剂(1.330 g, 10 mmol)。接下来,将混合物在70 ℃下磁力搅拌,并在N_2_保护下回流16 h。冷却至室温后,将所得白色沉淀物用二氯甲烷、甲醇和水彻底洗涤3次,然后在索氏提取器中用水-甲醇(1:1, v/v)进一步纯化24 h,以除去未反应的单体以及催化剂。最后,将得到的白色固体放入60 ℃真空干燥箱中干燥12 h,得到聚合物前体TP-TC。将0.20 g TP-TC和1.20 g MA分散在盛有50 mL二甲基亚砜的100 mL圆底烧瓶中,然后将混合物在160 ℃下磁力搅拌,并在N_2_保护下回流72 h。反应结束后,将沉淀物用二甲基亚砜、甲醇和水交替洗涤,而后用水-甲醇(1:1, v/v)通过索氏提取法进一步纯化24 h,以除去未反应的MA。最后,将该材料放入60 ℃真空干燥箱中干燥12 h,得到三聚氰胺功能化多孔有机聚合物TP-TC-MA。

### 1.3 甲基橙标准曲线绘制

首先配制100 mg/L的甲基橙母液,而后用超纯水稀释至0.1、1、5、10、20 mg/L,通过紫外-可见分光光度计(波长463 nm)测出吸光度,并以甲基橙的质量浓度为横坐标(*x*, mg/L),吸光度为纵坐标(*y*),绘制标准曲线。最终得到甲基橙溶液的标准曲线方程*y*=0.0421*x*-0.002,相关系数(*R*^2^)为0.999。

### 1.4 吸附实验

1.4.1 吸附动力学实验

将20 mg TP-TC-MA加入到20 mL初始浓度为100 mg/L的MO水溶液中,并调节溶液pH值至6.0,在间隔时间为0、2、5、10、20、30、60、120、180 min时取一定的溶液,通过紫外-可见分光光度计(波长:463 nm)测定每个时间点溶液中剩余MO的含量。TP-TC-MA对MO在时间*t*(min)下的吸附量*q_t_*(mg/g)由方程(1)计算:


(1) $q_{t}=\frac{\left(c_{0}-c_{t}\right) V}{m}$

式中,*c*_0_和*c_t_*分别表示吸附前和*t*时间时溶液中吸附质的质量浓度(mg/L);*V*表示溶液的总体积(L); *m*表示吸附剂的质量(g)。

1.4.2 吸附等温线实验

将20 mg TP-TC-MA分散于盛有20 mL MO水溶液的锥形瓶中,MO的初始浓度分别为50、100、200、300、400和500 mg/L,并调节溶液pH值至6.0。再将混合物在恒温水浴振荡器中于25 ℃下以160 r/min平衡2 h。最终将溶液在5000 r/min下离心5 min,取上清液稀释到一定倍数,经紫外-可见分光光度计测定吸附前后MO的浓度,并分别根据方程(2)、(3)计算平衡吸附量*q*_e_(mg/g)和去除率。


(2) $q_{\mathrm{e}}=\frac{\left(c_{0}-c_{\mathrm{e}}\right) V}{m}$

(3) $R=\frac{c_{0}-c_{\mathrm{e}}}{c_{0}} \times 100 \%$

式中, *c*_e_表示吸附平衡时溶液中吸附质的含量(mg/L)。

## 2 结果与讨论

### 2.1 三聚氰胺功能化多孔有机聚合物材料的结构性能表征

实验通过红外光谱来确定TP-TC-MA的特征官能团。如图1a所示,聚合物前体TP-TC在1644 cm^-1^处的峰归因于酮(C=O)的伸缩振动吸收,而在MA引入后,酮(C=O)的伸缩振动几乎消失。在3049、1470和1346 cm^-1^处出现的新的特征吸收峰分别归因于N-H、C=N和C-N的伸缩振动,表明MA已经成功对聚合物前体做了改性。通过X射线衍射分析材料的晶型结构,从图1b可以看出,TP-TC-MA和TP-TC的衍射图都存在一个宽峰,符合非晶态聚合物的典型特征。通过氮气吸附-脱附曲线来测试材料的比表面积和孔结构特征,结果如图1c所示。根据国际纯粹与应用化学联合会(IUPAC)的分类标准,TP-TC-MA的氮气吸附-脱附等温线属于典型的Ⅳ型,说明其具有介孔结构,而在低压区观察到氮气吸附量迅速上升说明其具有丰富的微孔(孔径分布见图1c的插图)。利用Brunauer-Emmet-Teller(BET)方法计算样品的BET表面积,结果表明经过MA改性后聚合物的比表面积和孔体积分别提高至708.5 m^2^/g和0.556 cm^3^/g。

**图1:**
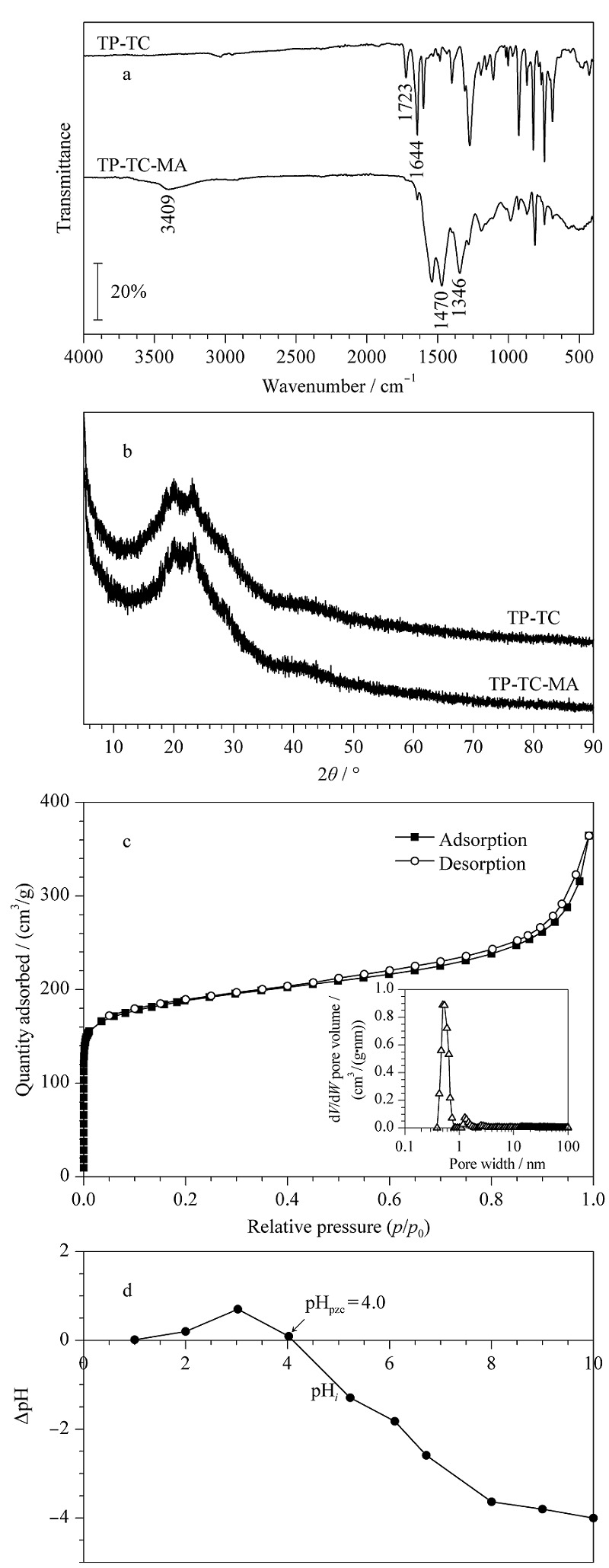
TP-TC-MA的表征

材料的零电荷点(pH_PZC_)是通过Balistrieri的方法以固体加入法测定的^[32]^。由图1d可知,随着起始pH的增加,ΔpH有一个先升高后下降的过程,TP-TC-MA的零电荷点在pH=4.0处,可能是因为材料存在较多的含氮官能团等碱性基团。

通过扫描电镜和透射电镜对所合成的多孔有机聚合物材料进行微观形貌表征,结果见图2。由扫描电镜图可知,经过MA改性后的聚合物显示出颗粒状聚集的形貌特征,在高分辨透射电镜图中也可以看出聚合物属于无定形结构,与X射线衍射图谱的表征结果一致。

**图2:**
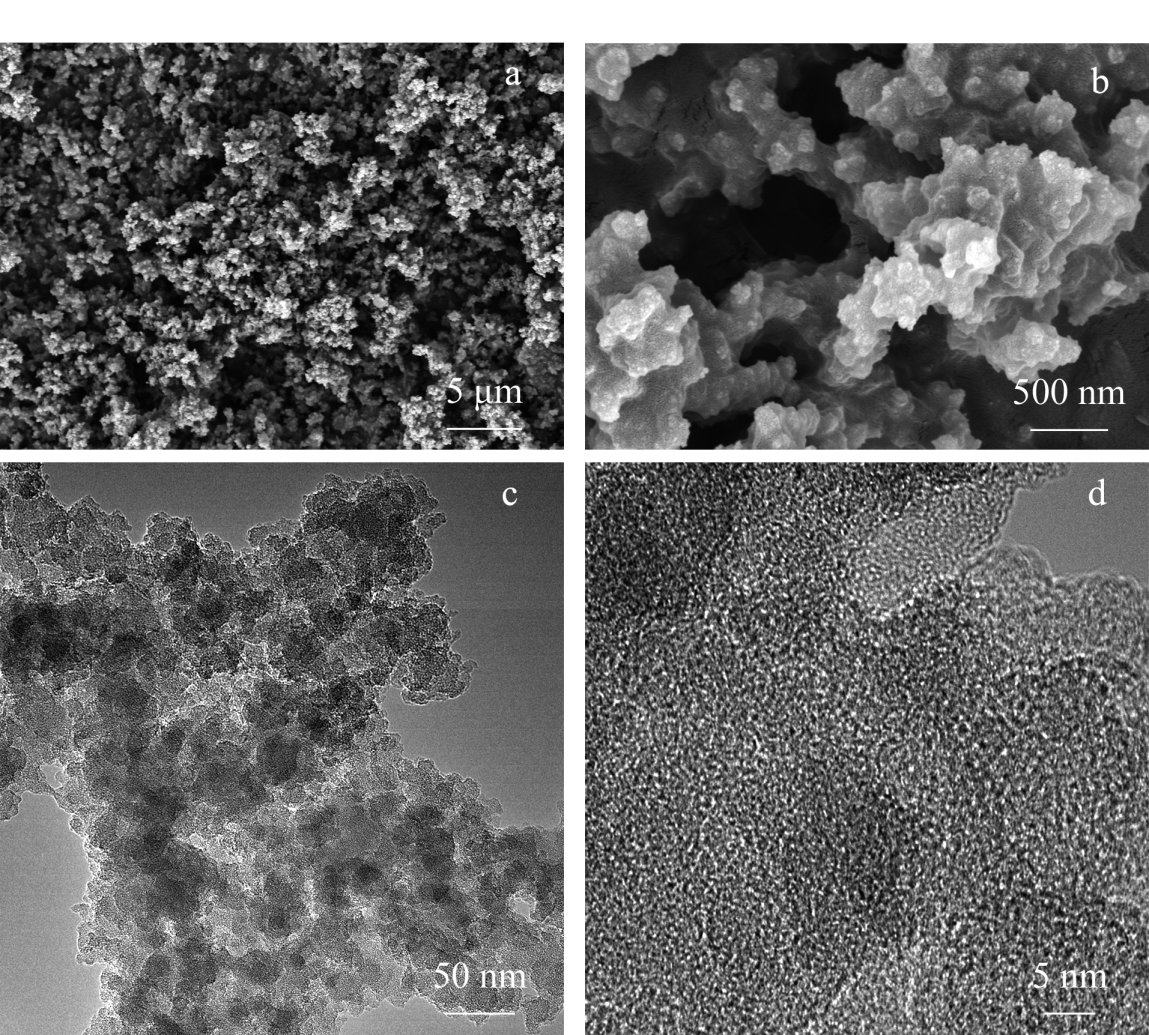
TP-TC-MA的微观形貌

这些结果说明三聚氰胺功能化多孔有机聚合物TP-TC-MA的成功合成。

### 2.2 吸附条件的考察

2.2.1 材料用量对吸附性能的影响

为了研究材料用量对吸附效果的影响,称取2、5、10、15、20和25 mg吸附剂分别加入到10 mL初始浓度为100 mg/L的MO溶液中,并调节溶液pH值为6.0,恒温振荡2 h后,离心分离。将上层清液稀释到一定倍数后,用紫外-可见分光光度计测定上清液中MO的浓度,并计算TP-TC-MA对MO的去除率,结果如图3a所示。当吸附剂的质量从2 mg增加到10 mg时,MO的去除率从57.5%增加到99.5%。这是因为随着吸附剂质量的增加,TP-TC-MA表面的吸附活性位点也相应增加,从而去除率有所提高。当吸附剂用量为10 mg时,MO的去除率已经接近最大值,继续增加吸附剂用量,去除率没有明显增加。因此,在溶液体积为 10 mL、初始浓度为100 mg/L的条件下,优化后的材料用量为10 mg。后续吸附实验保证材料用量和溶液体积比例一致。

**图3:**
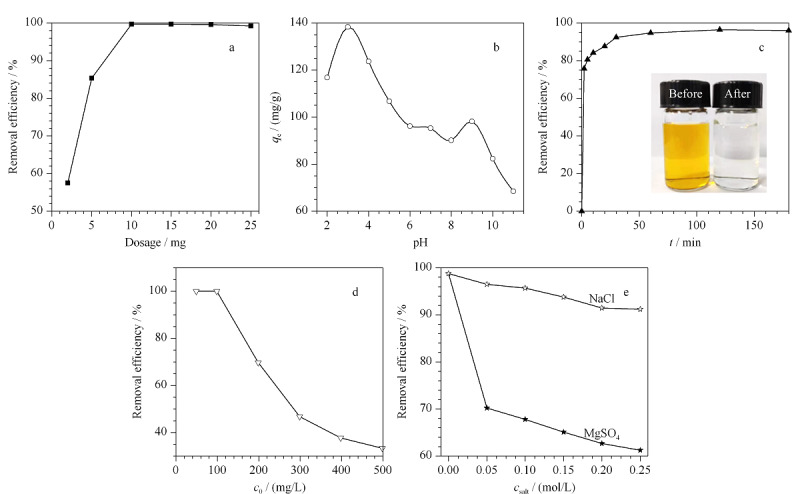
不同因素对TP-TC-MA吸附MO的影响

2.2.2 溶液初始pH的影响

吸附剂表面电荷和染料溶液化学性质的变化分别取决于零电荷点和酸解离常数(p*K*_a_)。通过考察TP-TC-MA对pH范围为2~11的100 mg/L MO溶液的吸附效果来评价溶液pH值对TP-TC-MA吸附MO的影响。结果如图3b所示,随着pH值的增加,MO的吸附容量先增加而后逐渐降低。这可以通过MO官能团所带的不同电荷与TP-TC-MA之间的静电吸引来解释。MO含有磺酸基(-$SO_3^-$)和胺基团(-N(CH_3_)_2_),其p*K*_a_为3.4,并且其存在形式受溶液pH值影响较大。在强酸体系中,溶液中的氢离子会与TP-TC-MA中的氨基和亚氨基产生质子化效应,有利于与阴离子染料MO产生静电引力,因此低pH值有利于阴离子染料MO的吸附。但pH过低时,溶液中的氢离子过多,MO中的氨基也会发生质子化反应,不利于MO与带正电荷的TP-TC-MA结合,同时多余的H^+^会与MO产生竞争吸附,不利于吸附过程,从而导致吸附剂对MO的吸附量下降;而在溶液pH值高于4时,氨基开始去质子化,而磺酸根的负电荷密度随pH值的增加而显著增加。pH值过高,质子化程度不足,同样会导致吸附容量下降。吸附剂的吸附效率也取决于其pH_pzc_,对于阳离子吸附质,在pH > pH_pzc_的溶液中观察到更高的吸附;然而,对于阴离子吸附质则相反。材料的pH_pzc_约为4.0,因此当pH高于4.0时,观察到材料的吸附性能有所下降。尽管材料在pH值为3.0的条件下对溶液中MO具有较高的吸附性能,但考虑到实际染料废水的pH值约为6~8,故本实验将溶液pH值条件为6.0。此外,pH过高时也发现材料对MO存在一定的吸附,说明材料对MO的吸附机理不仅仅存在静电相互作用,也可能存在氢键、芳香环的*π-π*共轭作用等。

2.2.3 吸附时间的影响

为了确定吸附达到平衡时所用的吸附时间,室温下在20 mL 100 mg/L MO的水溶液中加入20 mg TP-TC-MA,吸附剂对MO的去除率随时间的变化如图3c所示。在0~10 min时间段,因为有大量的活性位点被利用,短时间内MO的去除率迅速增加;吸附一段时间后,吸附位点逐渐饱和导致吸附缓慢,直至去除率不再增加。从图中可以看出,吸附过程在60 min时已逐渐达到吸附平衡。图3c插图显示了材料吸附前后MO溶液的颜色变化,即吸附开始前MO溶液为橙黄色,吸附180 min后溶液颜色基本消失,体现了材料对水中MO的去除能力。

2.2.4 溶液初始浓度的影响

在吸附过程中,溶液的初始浓度可以提供吸附推动力。一般说来,在一定浓度范围内,溶液初始浓度越大,则推动力越大,有利于快速吸附,但浓度过高会导致吸附不完全。

从图3d中可以看出,随着溶液初始浓度的增加,MO去除率逐渐降低。当MO的初始浓度在100 mg/L以下时,MO的去除率可以保持在99.9%以上,而当溶液初始浓度高于100 mg/L时,MO的去除率发生明显的下降。为实现较高的去除率,将MO溶液初始质量浓度定为100 mg/L。

2.2.5 离子强度的影响

由于实际废水中通常存在大量阴离子和阳离子,离子强度通过影响溶液中氢离子的分布,特别是被吸附离子的活度系数,从而影响吸附过程。因此,用不同浓度的盐溶液(NaCl、MgSO_4_)进一步考察离子强度对TP-TC-MA吸附MO的影响。由图3e的结果可以看出,不论是NaCl还是MgSO_4_都会对TP-TC-MA吸附MO产生抑制作用,可能是因为溶液中的阴离子(Cl^-^或$SO_{4}^{2-}$)占据活性位点,与MO产生竞争吸附,从而导致材料对MO的去除率降低。$SO_{4}^{2-}$比Cl^-^显示出更强的抑制作用,可能是因为$SO_{4}^{2-}$比Cl^-^带有较多的电荷,且离子尺寸较大。所以,较低的盐浓度有利于材料对MO的吸附。

### 2.3 吸附动力学

为考察TP-TC-MA对染料MO的吸附平衡时间,对吸附过程进行了吸附动力学研究,并采用准一级动力学模型和准二级动力学模型分析了MO在TP-TC-MA上吸附动力学特征,结果见图4。准二级动力学模型的拟合相关系数(*R*^2^=0.999)明显高于准一级动力学模型(*R*^2^=0.699),且计算得到的平衡吸附量*q*_e, cal_(94.34 mg/g)接近实验值*q*_e, exp_(93.69 mg/g)。动力学拟合的结果说明TP-TC-MA对MO的吸附过程遵循准二级动力学规律,吸附速率与MO浓度的二次方成正比,吸附剂的吸附性能与吸附剂存在的吸附位点成正比,且吸附过程以化学吸附为主。

**图4:**
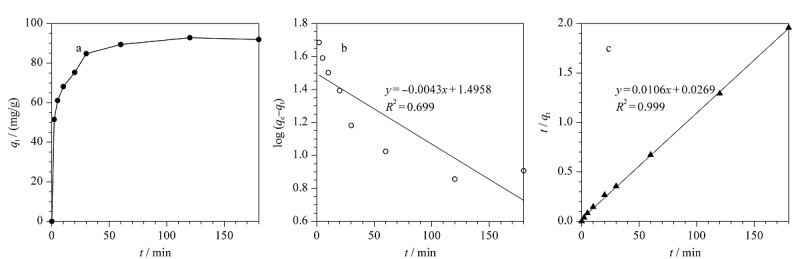
TP-TC-MA对MO的溶液的吸附动力学

### 2.4 吸附等温线

为了更充分地了解TP-TC-MA对MO的吸附行为特性,我们对吸附过程数据做了平衡吸附等温线研究。采用Langmuir和Freundlich模型对吸附数据进行线性拟合,结果如图5所示。可以看出,Langmuir模型拟合的*R*^2^(0.998)明显高于Frundlich模型的*R*^2^(0.798),说明材料对MO的吸附过程更符合Langmuir吸附模型,其吸附机理存在单层化学吸附过程。根据Langmuir模型计算得到的材料对MO的最大吸附量为156.3 mg/g,与实验值接近;而基于Frundlich模型的参数*n*表示吸附过程的支持力,1/*n*=0.107(介于0.1~0.5之间),说明吸附比较容易发生。

**图5:**
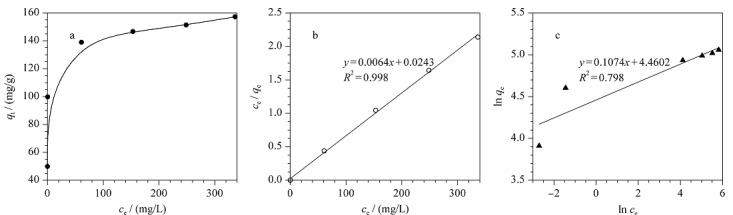
TP-TC-MA对MO的吸附平衡等温线

### 2.5 材料的重复利用性

为了考察TP-TC-MA对MO吸附的重复利用性,将10 mg吸附剂投入到10 mL 100 mg/L的MO溶液中并进行静态吸附2 h,使之吸附达到吸附平衡,而后用乙醇对吸附MO后的材料洗涤数次进行解吸附。如此循环5次来考察材料的重复利用性,结果如图6所示。随着使用次数的增加,材料对MO的去除效率逐渐降低。可能是因为在吸附-解吸附-再生的循环过程中,吸附剂与吸附质之间发生了化学反应,导致解吸不完全。重复使用5次之后,TP-TC-MA对MO的吸附去除率仍保持在90%以上,说明TP-TC-MA可多次循环利用。

**图6:**
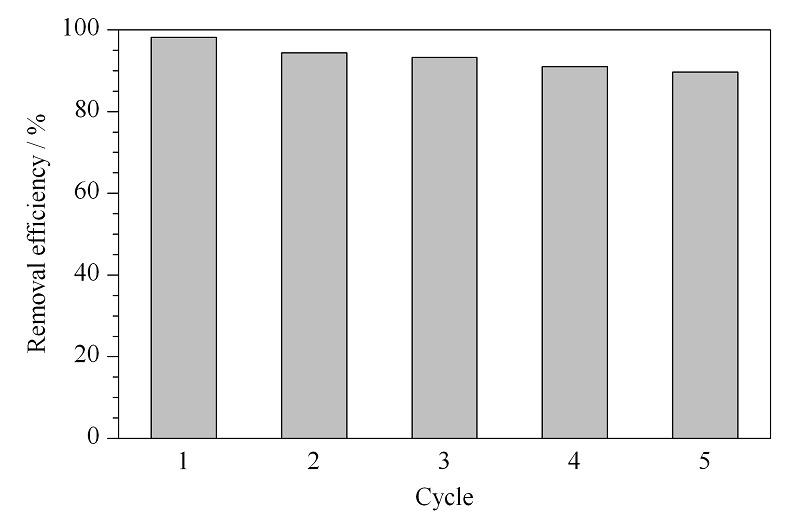
TP-TC-MA吸附MO的重复利用性

### 2.6 材料的选择性

为了进一步考察TP-TC-MA对MO的吸附选择性,将10 mg TP-TC-MA加入到MO和MB的混合溶液(20 mg/L)中,并用0.1 mol/L的HCl溶液(或0.1 mol/L的NaOH溶液)调节pH至3.0,恒温振荡一段时间,用紫外-可见分光光度计测得吸附前后溶液吸收光谱。从图7中可以看出,MO的吸光度几乎完全消失,但MB的吸收光谱只是略微有所降低。对比图7插图吸附前后溶液颜色从初始紫色变为蓝色,说明溶液中剩余染料大部分为MB。在酸性条件下,静电引力在吸附过程中起到很大作用,材料表现出对阴离子染料MO的特异性吸附。

**图7:**
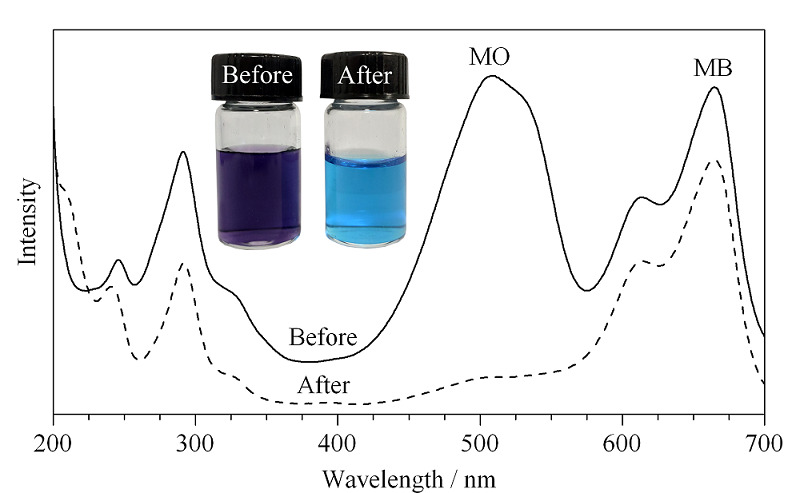
MO和MB的混合溶液经TP-TC-MA吸附前、 后的紫外-可见吸收光谱图

## 3 结论

本文通过两步法成功制备了三聚氰胺功能化的多孔有机聚合物TP-TC-MA,并将其用于水中阴离子染料甲基橙的吸附去除。TP-TC-MA具有很高的比表面积和孔体积以及丰富的含氮基团,对甲基橙表现出优异的吸附性能;稳定性好,可重复利用,从而实现染料废水的净化。本研究提出的三聚氰胺改性的多孔有机聚合物为处理染料废水提供了新的思路。
